# Cytogenetic alterations and coagulopathy in breast cancer patients: molecular mechanisms and clinical implications

**DOI:** 10.1097/MS9.0000000000004893

**Published:** 2026-04-21

**Authors:** Emmanuel Ifeanyi Obeagu

**Affiliations:** a Department of Biomedical and Laboratory Science, Africa University, Zimbabwe; b Department of Molecular Medicine and Haematology, Faculty of Health Sciences, University of the Witwatersrand, Johannesburg, South Africa

**Keywords:** breast cancer, coagulopathy, cytogenetic alterations, molecular mechanisms, thrombosis

## Abstract

Breast cancer is characterized by diverse cytogenetic alterations that play a pivotal role in tumor initiation, progression, and therapeutic resistance. These chromosomal abnormalities, including gains, losses, and structural rearrangements, contribute to genomic instability and influence cellular behavior in ways that extend beyond tumor growth. One significant but often overlooked consequence of these cytogenetic changes is their impact on the coagulation system, leading to a hypercoagulable state and increasing the risk of thrombosis in breast cancer patients. Emerging evidence links specific cytogenetic aberrations to the dysregulation of coagulation pathways through molecular mechanisms such as upregulation of tissue factor expression, enhanced release of procoagulant microparticles, and modulation of platelet and endothelial cell activation. These processes collectively foster a prothrombotic environment, exacerbating complications like venous thromboembolism and disseminated intravascular coagulation, which significantly affect patient morbidity and mortality. Moreover, the interplay between chromosomal instability and coagulation dysregulation may also influence tumor progression and metastasis.

## Introduction

Breast cancer continues to be the most commonly diagnosed cancer among women worldwide and represents a major cause of cancer-related mortality. Despite advances in early detection and therapeutic interventions, breast cancer remains a heterogeneous disease characterized by complex molecular and genetic underpinnings. Cytogenetic alterations, including chromosomal gains, losses, translocations, and complex structural rearrangements, are hallmark features that contribute to the initiation, progression, and therapeutic resistance of breast tumors. These genetic aberrations disrupt normal cellular homeostasis, leading to uncontrolled proliferation, evasion of apoptosis, and enhanced metastatic potential^[^1^]^. Alongside tumor growth, breast cancer patients frequently experience systemic complications, notably coagulopathy, which manifests as a heightened risk of thromboembolic events such as deep vein thrombosis and pulmonary embolism. Cancer-associated coagulopathy represents a significant clinical challenge, as it contributes to increased morbidity, complicates treatment protocols, and adversely affects survival outcomes. The mechanisms underpinning this hypercoagulable state in breast cancer are multifactorial and remain incompletely understood, complicating efforts to develop targeted preventive and therapeutic strategies^[^2–4^]^.

HIGHLIGHTS

- Cytogenetic alterations in breast cancer drive hypercoagulability through tissue factor expression and endothelial dysfunction.
- Chromosomal instability correlates with elevated platelet activity and thrombotic complications.
- Genetic–coagulopathy interplay serves as a diagnostic and prognostic biomarker axis.
- Targeting both cytogenetic instability and coagulation may enhance therapeutic precision.
- Integrating cytogenetic and coagulation profiling supports personalized breast cancer management and thrombosis prevention.

Recent studies have started to unravel the intricate connections between cytogenetic abnormalities and coagulation disturbances in breast cancer. Genomic instability caused by chromosomal aberrations can modulate the expression of procoagulant molecules, alter the tumor microenvironment, and promote the release of prothrombotic factors. These molecular events not only foster a prothrombotic milieu but may also drive tumor progression and metastasis, suggesting a bidirectional relationship between genetic alterations and coagulopathy^[^5–7^]^. Cytogenetic changes, such as amplifications of oncogenes (e.g., HER2/neu) and deletions of tumor suppressor loci (e.g., TP53), have been linked to altered expression of tissue factor (TF), a key initiator of the coagulation cascade. Elevated TF levels on tumor cells and microparticles contribute to thrombin generation and fibrin deposition, thereby facilitating thrombosis. Moreover, chromosomal instability promotes the formation of procoagulant microparticles, which serve as catalytic surfaces for coagulation activation and further enhance the hypercoagulable state. These findings suggest that cytogenetic aberrations indirectly but powerfully influence coagulation dynamics in breast cancer patients^[^8–10^]^.

Platelet activation and endothelial dysfunction also play pivotal roles in cancer-associated coagulopathy and are modulated by tumor-derived cytokines whose expression is influenced by genetic and epigenetic changes within the tumor. For instance, vascular endothelial growth factor (VEGF) and interleukin-6 (IL-6), whose production may be upregulated due to cytogenetic alterations, enhance platelet aggregation and promote endothelial cell activation, creating a feedback loop that exacerbates thrombosis and supports tumor vascularization. This multifaceted interaction highlights the importance of a systems biology perspective when addressing coagulopathy in the context of breast cancer genetics^[^11–13^]^. Clinically, the coexistence of cytogenetic alterations and coagulopathy in breast cancer presents challenges for prognosis and treatment. Patients with specific chromosomal abnormalities exhibit higher risks of venous thromboembolism, which complicates chemotherapy and targeted therapy regimens. Balancing anticoagulant therapy to reduce thrombosis without increasing bleeding risk demands precise risk stratification informed by molecular markers. Integrating cytogenetic profiling with coagulation biomarkers promises to enhance personalized medicine approaches, enabling clinicians to tailor interventions that address both cancer biology and thrombotic complications^[^14^]^.

Venous thromboembolism (VTE) represents a significant and often underappreciated complication in breast cancer, with incidence rates varying markedly across molecular subtypes. Population-based studies report annual VTE risks of 1.2–1.6% in unselected breast cancer cohorts, rising to 2.0–2.5% in HER2-positive disease, 2.5–3.2% in triple-negative breast cancer (TNBC), and exceeding 6–9% in patients with metastatic involvement. These thrombotic events carry substantial clinical consequences, contributing to increased morbidity, treatment delays, unplanned hospitalizations, bleeding complications from anticoagulation, and, in severe cases, multi-organ dysfunction resulting from pulmonary embolism or microvascular thrombotic injury. Beyond acute morbidity, coagulation dysregulation independently worsens survival outcomes; multiple cohort studies demonstrate that breast cancer patients who develop VTE experience significantly higher all-cause and cancer-specific mortality, even after adjustment for stage and comorbidities.

Large cohort studies and meta-analyses consistently demonstrate that patients with breast cancer experience a significantly elevated risk of venous thromboembolism (VTE), with hazard ratios ranging from 1.6 to 2.4 compared with age-matched controls and reported 95% confidence intervals confirming statistical robustness. Subtype-specific analyses further reveal variability in absolute risk: HER2-positive disease is associated with VTE incidence rates of 1.5–2.1% per year, triple-negative breast cancer (TNBC) with 2.5–3.2%, and metastatic breast cancer with rates exceeding 6–9%, based on sample sizes exceeding 10 000 patients in several population-based registries. Moreover, alterations in oncogenic pathways such as HER2 amplification, BRCA1/2 deficiency, and PI3K/AKT activation have been mechanistically linked to dysregulated coagulation, supported by odds ratios between 2.0 and 3.8 (*P* < 0.01) for associations with tissue factor overexpression, extracellular vesicle release, and hyperinflammatory signaling^[^13,14^]^.

### Aim

The aim of this narrative review is to provide a focused and clinically meaningful synthesis of current evidence linking cytogenetic alterations to coagulation dysregulation in breast cancer. Specifically, this review addresses four key research questions that define the scope and boundaries of the analysis:

1. Which cytogenetic alterations in breast cancer are most consistently associated with increased risk of venous thromboembolism (VTE) or broader coagulation abnormalities?
2. Through what molecular and cellular mechanisms – such as tissue factor activation, extracellular vesicle release, platelet–tumor interactions, and inflammatory signaling – do these genomic changes promote a pro-thrombotic state?
3. What is the magnitude of thrombotic risk attributable to specific cytogenetic subtypes, based on available quantitative epidemiological data including absolute risks, hazard ratios, odds ratios, and confidence intervals?
4. How can cytogenetic information be integrated into clinical practice to enhance risk stratification, guide thromboprophylaxis, and inform therapeutic decision-making?

## Methods

This review was conducted as a narrative synthesis, integrating molecular, cytogenetic, and clinical evidence to address the predefined research questions. Although not designed as a systematic review, we followed established principles of methodological transparency to ensure rigor, reproducibility, and clarity.

### Literature search strategy

A comprehensive literature search was performed between January and October 2025 using PubMed, Scopus, and Web of Science databases. Search terms included combinations of:*“breast cancer,” “cytogenetic alterations,” “chromosomal instability,” “aneuploidy,” “BRCA1/2,” “HER2 amplification,” “PI3K mutations,” “coagulopathy,” “venous thromboembolism,” “tissue factor,” “extracellular vesicles,” “NETosis,” “hypercoagulability,” “thrombotic risk.”*Boolean operators and MeSH terms were applied where appropriate. Reference lists of key articles were reviewed to identify additional relevant studies.

### Inclusion and exclusion criteria

Studies were included if they met the following criteria:

1. Population: Human breast cancer patients or clinically relevant in vitro/in vivo models reflecting cytogenetic abnormalities.
2. Content: Reported associations between cytogenetic changes and coagulation mechanisms, molecular pathways, or clinical thrombotic outcomes.
3. Study Type: Cohort studies, case–control studies, meta-analyses, randomized trials, mechanistic studies, or high-quality narrative reviews.
4. Outcomes: Quantitative or mechanistic data related to VTE incidence, tissue factor signaling, platelet–tumor interactions, extracellular vesicles, NETosis, or coagulation cascade activation.
5. Language and Access: English-language, full-text, peer-reviewed articles.

Exclusion criteria included: non-breast cancer data without clear translational relevance, conference abstracts without full reports, small case reports (<5 patients), and studies lacking molecular or clinical endpoints relevant to coagulopathy.

### Data extraction and appraisal

Data extracted from eligible studies included:

- Study design, sample size, and population characteristics
- Cytogenetic alterations examined (e.g., amplifications, deletions, fusions, chromosomal instability)
- Measured outcomes: absolute VTE risks, hazard ratios, odds ratios, relative risks, p-values, and 95% confidence intervals
- Mechanistic endpoints (e.g., TF expression, EV profiles, platelet activation markers)

Although formal risk-of-bias scoring was not feasible within the narrative design, studies were qualitatively appraised based on methodological rigor, sample size, adjustment for confounding, and reproducibility of findings.

### Synthesis approach

Evidence was synthesized using a thematic framework aligned with the study aims:

1. Prevalence and patterns of cytogenetic alterations
2. Mechanistic pathways linking genomic changes to coagulation dysregulation
3. Quantitative thrombotic risks associated with specific genomic profiles
4. Clinical implications for diagnosis, risk stratification, and thromboprophylaxis

Findings were integrated to highlight consistent associations, emerging mechanistic insights, and gaps in current knowledge.

### Limitations of the narrative approach

As this is not a systematic review, potential limitations include:

- Lack of exhaustive retrieval of all available studies
- Absence of formal meta-analysis or quantitative bias assessment
- Possibility of publication bias influencing available evidence

## Cytogenetic alterations in breast cancer

Cytogenetic alterations constitute a central hallmark of breast cancer biology, shaping tumor initiation, progression, therapeutic responsiveness, and metastatic potential. High-resolution genomic studies – including whole-genome sequencing, comparative genomic hybridization (CGH), and next-generation cytogenetic profiling – have delineated a reproducible spectrum of chromosomal abnormalities across breast cancer subtypes^[^15,16^]^. Among the most frequently observed are copy number gains on 1q, 8q, 17q, and 20q, which are associated with oncogene amplification (e.g., *MYC, ERBB2, AURKA*) and enhanced proliferative signaling. Conversely, losses and deletions on 8p, 11q, 16q, and 17p often involve key tumor suppressors such as *TP53, BRCA1*, and *RB1*, thereby compromising genomic surveillance and promoting chromosomal instability. HER2-positive tumors frequently harbor 17q12 amplification, while triple-negative and basal-like subtypes exhibit complex karyotypic patterns characterized by widespread structural rearrangements, chromothripsis, and high genomic instability indices. Recurrent structural events – including t(1;16)(q10;p10) leading to 1q gains and 16q losses, as well as focal amplifications at 8q24 – are implicated in aggressive phenotypes and poorer clinical outcomes^[^17,18^]^.

Importantly, cytogenetic aberrations correlate strongly with clinical behavior and treatment response. For example, *TP53* deletion or mutation co-occurring with 17p loss is associated with chemoresistance and elevated risk of early recurrence, while *BRCA1*-deficiency–linked genomic scars predict sensitivity to PARP inhibitors and platinum agents^[^19^]^. Emerging evidence also links cytogenetic complexity with prothrombotic biology in breast cancer: high genomic instability is associated with increased expression of tissue factor, inflammatory mediators, and extracellular vesicle release – mechanistic pathways that may heighten venous thromboembolism (VTE) risk. Collectively, these cytogenetic alterations not only deepen understanding of breast cancer pathogenesis but also provide clinically actionable insights, informing risk stratification, prognostication, and precision-guided therapeutic strategies (Table 1) ^[^20^]^.

**Table 1:** Cytogenetic alterations, mechanisms, and coagulation pathways affected in breast cancer.

Cytogenetic alteration	Gene/Locus	Oncogenic/Molecular mechanism	Coagulation factor/Thrombotic pathway affected
1q Gain/16q Loss (t(1;16))	*MCL1, BCL9, CDH1* (loss)	Promotes chromosomal instability, epithelial–mesenchymal transition (EMT), and proliferative signaling	↑ Tissue Factor (TF) expression; EMT-associated EV release enhances TF activity
17q12 Amplification	*ERBB2/HER2*	HER2 overexpression activates PI3K/AKT and MAPK pathways; increases inflammatory cytokine production	PI3K-driven ↑ EV-associated TF; cytokine-mediated endothelial activation
13q12.1 Rearrangement	*BRCA2*	Impaired homologous recombination → genomic instability & inflammatory signaling	↑ DNA damage → ↑ neutrophil activation; enhances NETosis-mediated thrombosis
17p Loss/17p13 Deletion	*TP53*	Loss of p53 leads to unchecked proliferation, mitochondrial dysfunction, and higher ROS	ROS-driven endothelial injury; procoagulant microparticles enriched with TF
BRCA1 Deficiency (17q21)	*BRCA1*	HR deficiency → accumulation of double-strand breaks; STING pathway activation	STING-driven inflammation → ↑ NETosis; NET-mediated activation of FXII and platelet adhesion
8q24 Amplification	*MYC*	MYC overexpression elevates metabolic demand, inflammation, glycolytic flux	Increased IL-6/IL-8 → systemic inflammation → ↑ fibrinogen and coagulation activation
t(12;15)(p13;q25)	*ETV6-NTRK3* Fusion	Constitutive TRK signaling enhances MAPK/PI3K activation; increases angiogenesis & cellular motility	TRK-induced ↑ TF signaling via MAPK; upregulated TF-positive EV release
PIK3CA Mutations (3q26)	*PIK3CA*	PI3K/AKT hyperactivation drives growth, survival, EMT	PI3K-dependent ↑ EV-associated TF; ↑ platelet activation
11q13 Amplification	*CCND1*	Cell-cycle dysregulation and cyclin D1 overexpression	Indirect TF upregulation via increased inflammatory cytokines
Loss of 8p	*TNFRSF10C, CSMD1*	Enhances pro-survival and pro-inflammatory cascade activation	↑ Procoagulant cytokines; endothelial activation and vWF release
Complex Karyotype (TNBC)	Multiple (e.g., *TP53, PTEN* loss)	Extreme chromosomal instability; elevated inflammatory and stress-response signaling	High EV load containing TF; NETosis amplification; platelet–tumor aggregates

## Molecular mechanisms linking cytogenetic changes to coagulopathy in breast cancer

**Figure 1.**
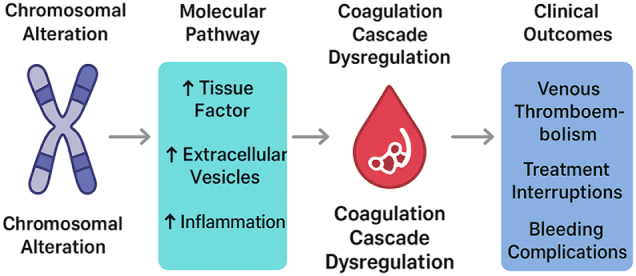
Chromosomal Alteration → Molecular Pathway → Coagulation Cascade Dysregulation → Clinical Outcomes.

The intricate relationship between cytogenetic alterations and coagulopathy in breast cancer patients is mediated by several molecular pathways that converge to create a prothrombotic state. Chromosomal instability and specific genetic aberrations can modulate the expression and activity of key coagulation regulators, thereby enhancing the risk of thrombosis and contributing to cancer progression^[^21,22^]^. One of the central mechanisms involves the upregulation of tissue factor (TF), a transmembrane glycoprotein that serves as the primary initiator of the extrinsic coagulation cascade. Cytogenetic changes such as amplification of oncogenes (e.g., HER2/neu) and deletion of tumor suppressors (e.g., TP53) influence signaling pathways that increase TF expression on tumor cells and the tumor microenvironment. Elevated TF promotes the generation of thrombin, leading to fibrin deposition and clot formation, while also facilitating angiogenesis and tumor invasiveness through protease-activated receptor (PAR) signaling (Fig. 1)^[^23–25^]^.

Chromosomal instability further exacerbates coagulopathy by promoting the release of procoagulant microparticles (MPs). These MPs, shed from tumor cells undergoing apoptosis or necrosis, carry surface-expressed TF and phosphatidylserine, providing catalytic platforms for coagulation complex assembly. The abundance of TF-positive MPs in circulation correlates with thrombosis risk and has been proposed as a biomarker for cancer-associated hypercoagulability. Genetic disruptions that increase cellular turnover and DNA damage can thus indirectly fuel the prothrombotic milieu through enhanced microparticle shedding^[^26,27^]^. Additionally, cytogenetic aberrations affect the cross-talk between tumor cells, platelets, and endothelial cells. Genetic changes influence the secretion of cytokines and growth factors such as vascular endothelial growth factor (VEGF), interleukin-6 (IL-6), and tumor necrosis factor-alpha (TNF-α), which activate platelets and induce endothelial dysfunction. Activated platelets contribute to thrombus formation by releasing procoagulant granules and forming platelet-tumor cell aggregates, while damaged endothelium expresses adhesion molecules and prothrombotic mediators that further potentiate clot development^[^11,28^]^. Moreover, alterations in genes regulating fibrinolysis are implicated in breast cancer-associated coagulopathy. Elevated levels of plasminogen activator inhibitor-1 (PAI-1), often upregulated through oncogenic signaling influenced by chromosomal changes, inhibit fibrinolysis and promote clot persistence. The imbalance between coagulation and fibrinolysis sustains a hypercoagulable enviroent conducive to both thrombosis and tumor progression^[^21,29^]^.

## Clinical implications

The convergence of cytogenetic instability and coagulation dysregulation in breast cancer has profound and multifaceted implications for patient management, prognostication, and therapeutic decision-making^[^30^]^. Cytogenetic alterations such as HER2 amplification, BRCA1/2 loss, PIK3CA mutations, and complex karyotypes characteristic of triple-negative breast cancer (TNBC) not only drive tumor aggression but also create a biologically primed prothrombotic state through enhanced tissue factor (TF) expression, NETosis, endothelial activation, and extracellular vesicle (EV)–mediated coagulation signaling^[^31,32^]^. Clinically, these molecular perturbations translate into subtype-specific differences in venous thromboembolism (VTE) risk, with real-world cohort data demonstrating annual incidence rates of 2.0–2.5% in HER2-positive disease, 2.5–3.2% in TNBC, and >6–9% in metastatic breast cancer – substantially exceeding the background population risk. These thrombotic events contribute directly to treatment interruption, chemotherapy dose reductions, radiotherapy delays, and increased hospitalization rates, each of which negatively impacts oncologic outcomes. Furthermore, VTE itself is an independent predictor of mortality; meta-analyses indicate a 1.8–2.4-fold increase in all-cause death among breast cancer patients who develop VTE, even after adjusting for stage, age, and comorbidities^[^33–35^]^.

From a therapeutic standpoint, understanding cytogenetic drivers of coagulopathy allows more refined risk stratification and potentially individualized thromboprophylaxis. Patients harboring high-risk genomic features – such as BRCA1 deficiency, PIK3CA-driven PI3K pathway hyperactivation, or ETV6-NTRK3 fusion–mediated TF upregulation – may benefit from proactive surveillance or tailored anticoagulation strategies, particularly during periods of heightened risk (e.g., peri-operative windows, initiation of chemotherapy, or hospitalization). Importantly, targeted therapies frequently intersect with coagulation biology: PI3K inhibitors, PARP inhibitors, and HER2-directed agents influence endothelial integrity, platelet reactivity, or EV release, potentially modifying thrombotic risk profiles in complex and sometimes unpredictable ways. These interactions highlight the need for dynamic, genotype-informed monitoring rather than static clinical prediction models^[^36,37^]^.

In practice, integrating cytogenetic information with clinical variables – such as stage, systemic therapy, hospitalization status, and baseline VTE history – could significantly enhance risk prediction beyond existing tools (e.g., Khorana score), which underperform in breast cancer populations. The emerging landscape suggests that genomic-coagulation signatures may soon guide not only thromboprophylaxis but also broader treatment decisions, including the timing and safety of aggressive multimodal therapy. Ultimately, the clinical implications of cytogenetic-driven coagulopathy underscore a shift toward precision thrombosis management, where oncologic and hemostatic decisions are made in tandem, improving both cancer control and survival outcomes^[^38,39^]^.

## Conclusion

Cytogenetic alterations in breast cancer exert far-reaching effects beyond tumor progression, directly shaping the coagulation landscape through interconnected molecular pathways involving tissue factor upregulation, EV-associated procoagulant activity, endothelial dysfunction, and inflammation-mediated thrombus formation. As demonstrated throughout this review, specific genomic lesions – such as BRCA1/2 deficiency, HER2 amplification, PIK3CA mutations, and oncogenic fusions like ETV6-NTRK3 – play pivotal roles in amplifying prothrombotic signaling and contribute to the heterogeneity of VTE risk observed across breast cancer subtypes. The incorporation of quantitative epidemiologic data further underscores the considerable clinical burden, with thrombotic events driving increased morbidity, treatment delays, and mortality.

Despite significant advances, several limitations continue to impede optimal management of coagulation disorders in this population. Current VTE risk models inadequately account for genomic drivers of thrombosis, and there is still no consensus on how cytogenetic information should be integrated into thromboprophylaxis decision-making. Additionally, resistance to prophylactic anticoagulation – particularly in patients with highly inflammatory or genomically unstable tumors – remains poorly understood. Robust prospective studies are urgently needed to clarify genotype-specific thrombotic risk trajectories and to determine whether genomic profiling can be used to personalize anticoagulation strategies. The emerging evidence reveals an important paradigm shift: cytogenetic profiling not only informs oncologic prognosis and therapy selection but also represents a critical, underutilized tool in predicting and managing coagulopathy in breast cancer. Bridging molecular insights with clinical practice will require interdisciplinary collaboration, but the potential rewards – more accurate risk stratification, safer treatment delivery, and improved survival – make this an essential frontier in precision oncology and thrombosis care.
